# miR-30a as Potential Therapeutics by Targeting TET1 through Regulation of Drp-1 Promoter Hydroxymethylation in Idiopathic Pulmonary Fibrosis

**DOI:** 10.3390/ijms18030633

**Published:** 2017-03-15

**Authors:** Songzi Zhang, Huizhu Liu, Yuxia Liu, Jie Zhang, Hongbo Li, Weili Liu, Guohong Cao, Pan Xv, Jinjin Zhang, Changjun Lv, Xiaodong Song

**Affiliations:** 1 Department of Cellular and Genetic Medicine, School of Pharmaceutical Sciences, Binzhou Medical University, Yantai 264003, China; 13954359981@163.com (S.Z.); 18865675850@163.com (H.L.); yimeng3183@163.com (Y.L.); 18547131029@163.com (J.Z.); jjinzhang@126.com (J.Z.); 2 Department of Clinical Pharmacology, School of Pharmaceutical Sciences, Taishan Medical University, Taishan 271016, China; 3 Department of Respiratory Medicine, Affiliated Hospital to Binzhou Medical University, Binzhou 256602, China; lihongbo0516@sina.com (H.L.); lwl249880849@163.com (W.L.); cgh861025@126.com (G.C.); xupan_333@126.com (P.X.)

**Keywords:** idiopathic pulmonary fibrosis, miR-30a, TET1, Drp-1

## Abstract

Several recent studies have indicated that miR-30a plays critical roles in various biological processes and diseases. However, the mechanism of miR-30a participation in idiopathic pulmonary fibrosis (IPF) regulation is ambiguous. Our previous study demonstrated that miR-30a may function as a novel therapeutic target for lung fibrosis by blocking mitochondrial fission, which is dependent on dynamin-related protein1 (Drp-1). However, the regulatory mechanism between miR-30a and Drp-1 is yet to be investigated. Additionally, whether miR-30a can act as a potential therapeutic has not been verified in vivo. In this study, the miR-30a expression in IPF patients was evaluated. Computational analysis and a dual-luciferase reporter assay system were used to identify the target gene of miR-30a, and cell transfection was utilized to confirm this relationship. Ten–eleven translocation 1 (TET1) was validated as a direct target of miR-30a, and miR-30a mimic and inhibitor transfection significantly reduced and increased the TET1 protein expression, respectively. Further experimentation verified that the TET1 siRNA interference could inhibit Drp-1 promoter hydroxymethylation. Finally, miR-30a agomir was designed and applied to identify and validate the therapeutic effect of miR-30a in vivo. Our study demonstrated that miR-30a could inhibit TET1 expression through base pairing with complementary sites in the 3′untranslated region to regulate Drp-1 promoter hydroxymethylation. Furthermore, miR-30a could act as a potential therapeutic target for IPF.

## 1. Introduction

Idiopathic pulmonary fibrosis (IPF) is a chronic, progressive, and lethal fibrotic lung disease [1]. IPF is characterized by alveolar epithelial cell injury and activation, myofibroblast foci formation, and exaggerated extracellular matrix accumulation in the lung parenchyma. Despite significant progress, the etiology and molecular mechanisms underlying IPF are largely unknown [2].

MicroRNAs (miRNAs) constitute a large family of regulatory RNAs that inhibit target expression through base pairing with complementary sites in the 3′ untranslated region (3′UTR) to promote mRNA decay and translational repression [3]. The critical roles of miRNAs in development and the mechanism underlying disease occurrence associated with their dysregulation have become increasingly known [4]. Therefore, identifying disease-specific miRNAs can reveal novel mRNA target pathways and provide insight into the pathogenesis of specific diseases and therapeutic targets [5]. Recent research reported that circulating miRNAs are very stable in plasma and serum and remain stable when free from RNases under conditions of long-term storage or repeated freezing and thawing; these features show that circulating miRNAs from patients can be used as a biomarker for diagnosis and treatment [6,7,8]. However, the mechanisms regarding the control of IPF progression by circulating miRNAs remain poorly understood.

Oxidative stress is an important phenomenon in pathological IPF processes and is commonly associated with the oxidative damage of epithelial cells, which is believed to be the initial event that triggers a series of aberrant repair pathways that lead to inappropriate fibrosis [9]. Du Bois once proposed that targeting the manipulation of epithelial cell apoptosis appears to be an advantageous method to treating IPF, considering that novel antioxidant approaches are also recommended [10]. Therefore, our study focused on miR-30a regulation on the epithelial cell apoptosis during H_2_O_2_-induced IPF. Pandit et al. [11] reported that the miR-30 family is significantly decreased in IPF patients. However, the regulatory mechanism of miR-30 in IPF has never been systematically explored until now. Our previous study demonstrated that miR-30a may function as a novel therapeutic target for lung fibrosis by blocking mitochondrial fission, which is dependent on dynamin-related protein1 (Drp-1) [12]. However, three critical issues must be addressed. First, miR-30a expression in IPF patients has not been evaluated. Second, the target gene of miR-30a and the regulatory mode between the target gene and Drp-1 remain to be investigated. Third, whether miR-30a can act as a potential therapeutic has not been verified in vivo. In this study, we further evaluated the circulating miR-30a expression in IPF patients and explored the miR-30a target gene of methylcytosine dioxygenase ten–eleven translocation 1 (TET1) and the regulatory mode between TET1 and Drp-1. Finally, we designed and applied the miR-30a agomir to identify and validate the therapeutic effect of miR-30a in vivo.

## 2. Results

### 2.1. miR-30a Expression in Patients and Analysis of the Possible Target Gene

**Figure 1:**
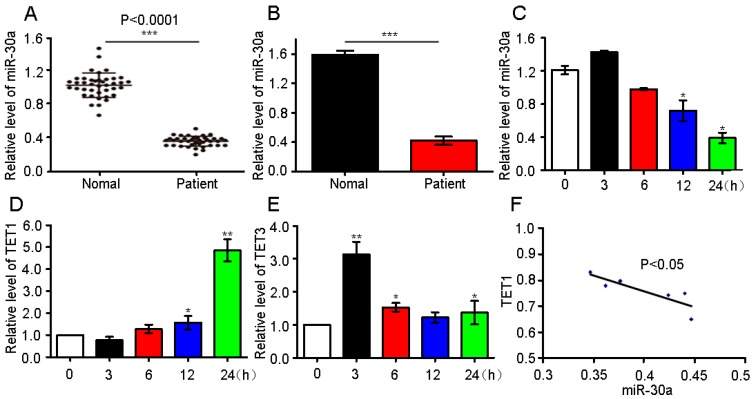
miR-30a expression in idiopathic pulmonary fibrosis (IPF) patient and analysis of the possible target gene. (**A**) Forty patients with IPF had decreased circulating miR-30a compared with normal individuals based on the quantitative real-time polymerase chain reaction (qRT-PCR) analysis. U6 served as internal control; (**B**) Six patients with IPF had decreased circulating miR-30a compared with normal individuals based on the qRT-PCR analysis. Cel-miR-39-3p served as an external control; (**C**) The qRT-PCR analysis showed that miR-30a was decreased in MRC-5 treated with H_2_O_2_. Human fetal lung fibroblast MRC-5 cell was treated with 90 µM H_2_O_2_ and harvested at 0, 3, 6, 12, and 24 h. The data of each group are presented as mean ± SD, *n* = 6, * *p* < 0.05. U6 served as an internal control; (**D**) The qRT-PCR analysis showed that ten–eleven translocation 1(TET1) increased with the time extension in the H_2_O_2_-treated group. A549 was treated with 120 µM H_2_O_2_ and harvested at 0, 3, 6, 12, and 24 h. The data of each group are presented as mean ± SD, *n* = 6, * *p* < 0.05, ** *p* < 0.01. U6 served as an internal control; (**E**) TET3 showed a disordered expression with the time extension in the H_2_O_2_-treated group. A549 was treated with 120 µM H_2_O_2_ and harvested at 0, 3, 6, 12, and 24 h. The data of each group are presented as mean ± SD, *n* = 6, * *p* < 0.05, ** *p* < 0.01; (**F**) miR-30a is inversely correlated with TET1. Statistical analysis was conducted using Pearson’s correlation coefficient, *r* = −0.8508, *p* = 0.0317.

Our previous study reported that miR-30a expression is decreased in the in vivo and in vitro lung fibrosis models [12]. In this study, we further evaluated the miR-30a expression in IPF patients to assess the significance of miR-30a in clinical research. Characteristics and physiologies of IPF patients and the normal individuals are shown in Table 1. The quantitative real-time polymerase chain reaction (qRT-PCR) result shows that the circulating miR-30a expression level is lower in IPF patients than those in normal individuals (Figure 1A,B).To confirm miR-30a expression, we further tested it in MRC-5 cells, which are derived from human embryonic lung fibroblast cells (Figure 1C). All results are in agreement with those of our previous work with in vivo and in vitro models [12].

miRNAs exert their regulatory functions through specific interactions with their target genes. Therefore, the target genes of miR-30a were first predicted based on TargetScan, miRanda data, and miRbase. Our previous studies show that miR-30a inhibits AECII apoptosis by repressing the mitochondrial fission dependent on Drp-1 to block IPF progress [12]. Therefore, we speculate that Drp-1 may be the target gene of miR-30a. However, the computational analysis data showed that no binding sites with miR-30a were detected in the 3′UTR of Drp-1. Moreover, the computational analysis data suggested that binding sites with miR-30a may exist in the 3′UTR of TET1 and TET3. Thus, TET1 and TET3 are the potential candidate target genes. qRT-PCR was used to further identify TET1 and TET3 as possible miR-30a targets. The results indicate that TET1 expression increased with the increase in H_2_O_2_ treatment time, suggesting the time dependence of the TET1 expression (Figure 1D). Meanwhile, TET3 expression displayed a disordered condition (Figure 1E). Further analysis of the Pearson’s correlation coefficient demonstrated that miR-30a is inversely correlated with TET1 (Figure 1F). Therefore, only TET1 was selected as an miR-30a target for further study.

### 2.2. Confirmation of Ten–Eleven Translocation 1(TET1) as a Target Gene of miR-30a

**Figure 2:**
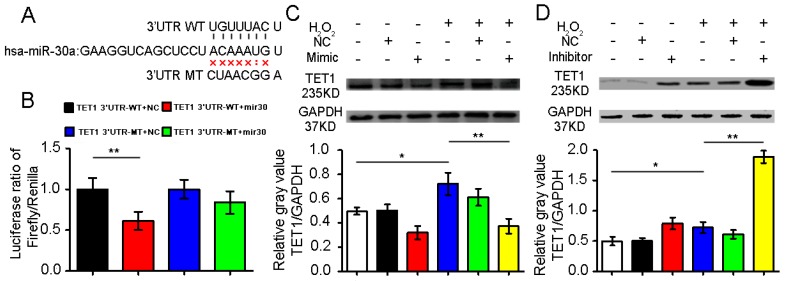
Experimental confirmation of TET1 as a target gene of miR-30a. (**A**) Binding site with miR-30a in 3′UTR of TET1. WT, wild type; MT, mutation type; (**B**) Ratios of firefly/*Renilla* in the dual-luciferase reporter system analysis. The luciferase ratio of firefly/*Renilla* represents the target gene expression. The luciferase activity of the WT 3′UTR-TET1 significantly decreased in the cells transfected with miR-30a mimic. However, the miR-30a mimic could not inhibit the luciferase activities of the MT 3′UTR-TET1; (**C**) The miR-30a mimic inhibited the expression of TET1 in the A549 cells treated with H_2_O_2_ for 24 h. NC is the negative control of the miR-30a mimic; (**D**) The miR-30a inhibitor improved TET1 expression in the A549 cells treated with H_2_O_2_ for 24 h. NC is the negative control of the miR-30a inhibitor. +/− Indicates with or without reagent respectively.The data of each group are presented as mean ± standard deviation (SD), * *p* < 0.05, ** *p* < 0.01.

To verify if TET1 is a target gene of miR-30a, we set up a dual-luciferase assay reporter system by amplifying and inserting the 3′UTR of TET1 into the pMIR-REPORT vector, which contained downstream firefly luciferase. The luciferase activity of the wild-type (WT) 3′UTR-TET1 was significantly decreased in the cells transfected with the miR-30a mimic, which could not inhibit the luciferase activities of the mutant-type (MT) 3′UTR-TET1 (Figure 2A,B). The data suggest that TET1 may be a miR-30a target.

To confirm whether TET1 is a target gene of miR-30a, we used cell transfection with miR-30a mimic or inhibitor to identify the relationship. The H_2_O_2_-treated group had increased TET1 expression levels compared with the control group. Additionally, the miR-30a mimic could inhibit TET1 expression, whereas the miR-30a inhibitor could improve the TET1 expression (Figure 2C,D). The data further confirm that TET1 is a target gene of miR-30a.

### 2.3. TET1 Interference Inhibited Dynamin-Related Protein1 (Drp-1) Promoter Hydroxymethylation

**Figure 3:**
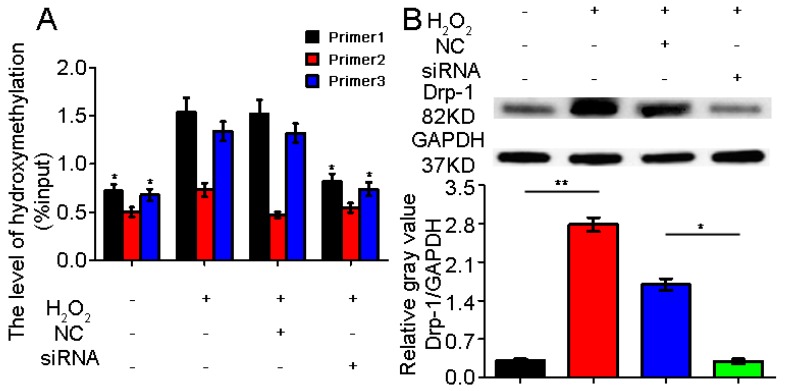
Effect of TET1 siRNA on the hydroxymethlation of the dynamin-related protein1 (Drp-1) promoter. (**A**) TET1 siRNA could inhibit the hydroxymethlation expression of the Drp-1 promoter compared with the H_2_O_2_ group; (**B**) The Drp-1 expression decreased significantly after TET1 siRNA. NC is the negative control of TET1 siRNA. +/− Indicates with or without reagent respectively. The data of each group are presented as means ± SD, * *p* < 0.05, ** *p* < 0.01.

Our previous study showed that miR-30a could block the pulmonary epithelial cell apoptosis by repressing mitochondrial fission, which is dependent on Drp-1. However, the regulatory mode between TET1 and Drp-1 remains to be determined. We further explored the regulated pattern between TET1 and Drp-1. We designed three specific primers for the predicted methylation sites in the Drp-1 promoter region and interference on TET1 siRNA. The results of the hydroxymethylation experiment demonstrated that Drp-1 expression hydroxymethylation increased in the A549 cells treated with H_2_O_2_ for 24 h. However, Drp-1 expression hydroxymethylation decreased in the A549 cells treated with H_2_O_2_ for 24 h following treatment with TET1 siRNA. TET1 interference could significantly inhibit Drp-1 expression hydroxymethylation (Figure 3). Mutual regulation may be correlated with the hydroxymethylation mode between TET1 and Drp-1. Meanwhile, the expression of the tested Drp-1 significantly decreased after TET1 interference.

### 2.4. miR-30a as a Potential Therapeutic Target for Idiopathic Pulmonary Fibrosis (IPF)

**Figure 4:**
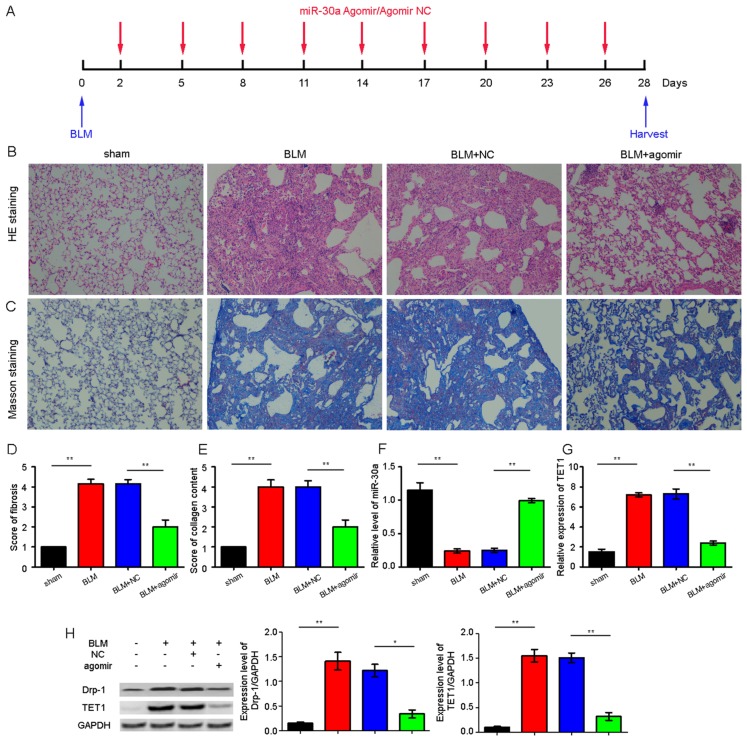
miR-30a as a potential therapeutic target for IPF. (**A**) Schematic of the miR-30a agomir action; (**B**) miR-30a agomir could improve the alveolar structure in vivo through hematoxylin and eosin (H&E)staining, ×400 magnification; (**C**) miR-30a agomir could inhibit the collagen fibers through Masson’s staining. The color blue represents collagen fibers, ×400 magnification; (**D**) Score of lung fibrosis on H&E staining; (**E**) Score of collagen content on Masson’s staining; (**F**) Mice sprayed with agomir had increased miR-30a expression compared with those in the bleomycin (BLM)group based on qRT-PCR; (**G**) Mice sprayed with agomir had decreased TET1 expression compared with those in the BLM group based on qRT-PCR; (**H**) Mice sprayed with agomir had decreased Drp-1 and TET1 expression compared with those in the BLM group based on the Western blot. +/− Indicates with or without reagent respectively. Each bar represents the mean ± SD, *n* = 6, * *p* < 0.05, ** *p* < 0.01.

To explore whether miR-30a can act as a potential therapeutic in vivo, we synthesized a miR-30a agomir and sprayed it across the mouse lung using a Penn-Century MicroSprayer (Penn-Century Inc., Wyndmoor, PA, USA). The fibrosis and the collagen content were evaluated using hematoxylin and eosin (H&E) and Masson’s staining. The mice in the miR-30a agomir group presented a more continuous bronchial mucous membrane structure with a better intact wall than those in the bleomycin (BLM) group. The alveoli in the miR-30a agomir group showed clearer hollow cavities with thinner alveolar walls than those in the BLM group. Additionally, the lung mesenchyme in the miR-30a agomir group displayed fewer collagen fibers, thereby indicating that the hallmark of the fibroblastic foci was distinctly decreased (Figure 4A–E). The miR-30a agomir could reduce pulmonary fibrosis. The BLM group had significantly decreased miR-30a level and significantly increased TET1 level compared with the sham group; furthermore, miR-30a agomir could improve the miR-30a expression and inhibit the TET1 expression in the agomir group compared with the BLM group (Figure 4F–H).

**Figure 5:**
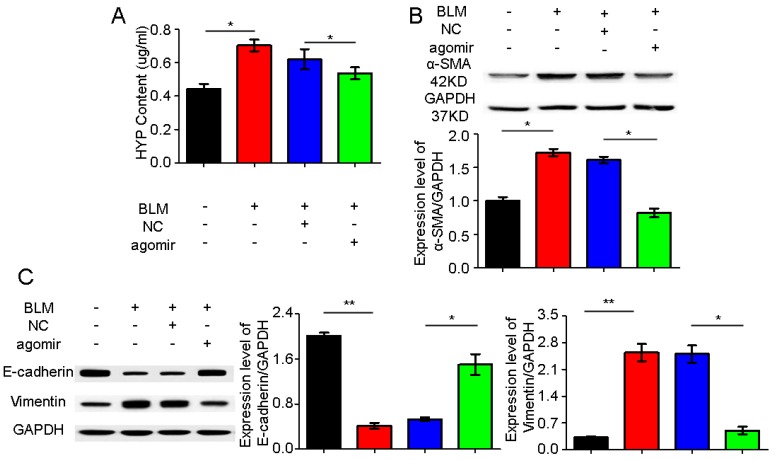
miR-30a agomir inhibited the indicators of pulmonary fibrosis in vivo. (**A**) miR-30a agomir could inhibit the HYP level; (**B**) miR-30a agomir could inhibit the a-SMA expression; (**C**) miR-30a agomir could promote the E-cad expression and inhibit the vimentin expression. +/− Indicates with or without reagent respectively.Each bar represents the mean ± SD, *n* = 6, * *p* < 0.05, ** *p* < 0.01.

To further investigate the antifibrotic action of miR-30a agomir in vivo, we tested the expression levels of hydroxyproline (HYP), a smooth muscle actin (a-SMA), *E-cadherin*, and vimentin, which are indicators of pulmonary fibrosis, after miR-30a agomir spraying in vivo. The agomir could decrease the HYP, a-SMA, and vimentin expression levels but increase the *E-cadherin* expression in the miR-30a agomir group compared with the BLM group (Figure 5).

## 3. Discussion

Currently, IPF is the most life-threatening idiopathic disease, presenting a mortality rate that exceeds those of numerous cancers [10,13]. Although several genetic, epigenetic, and proteomic studies have been conducted to date, studies investigating miRNA regulatory networks in IPF have only recently gained significant attention [2]. Our previous study revealed that miR-30a can block mitochondrial fission, which is dependent on Drp-1, to regulate the IPF development. However, the regulatory mechanism between miR-30a and Drp-1 remains to be investigated. The miR-30 family includes miR-30a, miR-30b, miR-30c, miR-30d, and miR-30e and serves important functions in various life activities and illness developments, such as epithelial-to-mesenchymal transition, cellular differentiation and senescence, and cancer [14,15]. The family members share the same seed sequence and are encoded by six genes located in human chromosomes 1, 6, and 8. The genes identified as targets of one or more miR-30 family members include Xlim1/Lhx1 [16], Snail1 [17], BCL6 [18], p53 [19], and RUNX2 [20]. In the present study, we identified and validated TET1 as a target of miR-30a and the regulatory mode between TET1 and Drp-1. Furthermore, we demonstrated that miR-30a could act as a potential therapeutic target by targeting TET1 through regulation of the Drp-1 promoter hydroxymethylation in IPF.

The TET protein family includes three members (TET1–TET3), all of which are capable of converting 5-methylcytosine to 5-hydroxymethylcytosine (5-hmC) in a 2-oxoglutarate- and Fe(II)-dependent manner [21,22]. Additionally, DNA methylation at the 5-position of cytosine is a key epigenetic mark, which is critical for various biological and pathological processes [23,24]. Genome-wide studies demonstrated the 5-hmC enrichment at the enhancers, promoters, and gene bodies of actively expressed genes [25,26]. The presence of 5-hmC may contribute to both passive and active DNA demethylation processes. TET1 is required in maintaining normal abundance and distribution of 5-hmC, which prevents DNA hypermethylation and promotes DNA demethylation through a sequential process involving the deamination of 5-hmC to 5-hmU (from C to U transitions); moreover, TET1 is required for the regulation of gene-encoding molecules involved in chromosome maintenance and DNA repair [27]. TET1 expression is the highest in long-term hematopoietic stem cells (HSCs), multipotent progenitors, common lymphoid progenitors, and common myeloid progenitors; decreases during B-lineage commitment; and becomes undetectable in immature and mature myeloid cells. The loss of TET1 function predisposes HSCs to malignancy and, more specifically, B-cell lymphoma [28]. In drug addiction, TET1 normally regulates cocaine reward negatively, and the cocaine-induced suppression of TET1 in nucleus accumbens contributes to enhanced drug sensitivity [29]. This epigenetic lesion has also been extended to the DNA methylation-mediated silencing of miRNA [30,31,32]. miR-22 was recently found to exert its metastatic potential by silencing antimetastatic miR-200 through the direct targeting of the TET family of methylcytosine dioxygenases, thereby inhibiting the miR-200 promoter demethylation [33]. However, the mechanism by which this epigenetic 5-hmC mark and TET-related enzyme participate in IPF progression remains unknown. The decrease in the5-hmC of Drp-1 was caused, at least in part, by the decreased expression level of the key enzyme TET1, which controls the5-hmC production.

Several recent studies indicated that miR-30a performs critical roles in various biological processes. For example, Ubc9 (ubiquitin carrier protein 9) expression is regulated by miR-30a in human subcutaneous adipocytes [34]. miR-30a reduces IRF4 expression through specific binding with the 3′UTR, thereby suppressing the Th17 differentiation and preventing the full autoimmune encephalomyelitis development [35]. miR-30a targets the DNA replication protein RPA1, hinders DNA replication, and induces DNA fragmentation [36]. These studies lack information on the therapeutic effect of miR-30a in vivo. Moreover, data on pulmonary fibrosis data are yet to be reported. To further verify the therapeutic effect of miR-30a, the miR-30a agomir was designed and applied in vivo. Here, we found that injection of miR-30a agomir to overexpressed miR-30a leads to thinner alveoli walls and fewer collagen fibers, which could speed up the diffusion of oxygen into the adjacent blood capillaries and the opposite movement of carbon dioxide compared with the BLM group.

We assessed the significance of circulating miR-30a in clinical application. Future studies need to expand the analysis of miR-30a to a larger cohort of patients with IPF to determine with statistical confidence whether reduced miR-30a expression levels are correlated with poor patient outcome. In summary, our in vivo and in vitro findings, together with the clinical data of patients with IPF, provide evidence that miR-30a upregulation could downregulate the expression level of its target gene TET1 and exert antifibrotic effect and protective function against pulmonary injury. Our work suggests a novel approach to prevent pulmonary fibrosis damage and provides a potential therapeutic target for pulmonary fibrosis treatment.

## 4. Materials and Methods

### 4.1. IPF Patients

IPF was diagnosed based on the American Thoracic Society/European Respiratory Society consensus criteria [1], which include clinical, radiographic, and characteristic histopathological features (*n* = 46). A 5 mL blood sample was obtained from each participant and prepared for testing. Matched plasma samples from healthy volunteers (*n* = 46) were selected corresponding to the IPF patients’ sex and age. As stated in the agreement, a written informed consent was obtained by the doctors from each participant. The Ethics Committee of Binzhou Medical University approved this study.

### 4.2. Human Fetal Lung Fibroblast MRC-5 Cell Culture

MRC-5 cell lines were purchased from the Cell Bank of the Chinese Academy of Sciences (Shanghai, China) and maintained in advanced minimum essential medium supplemented with 10% fetal bovine serum, 2% penicillin, and streptomycin at 37 °C and 5% atmosphere. MRC-5 cells were used for experiments at an initial density of 70%–80%.

### 4.3. Cell Transfection

A549 cell lines were purchased from the Cell Bank of the Chinese Academy of Sciences (Shanghai, China). Cell culture was prepared as previously described [37,38]. miR-30a mimic and inhibitor and TET1 siRNA were synthesized by RiboBio Co., Ltd. (Guangzhou, China). A total of 1 × 10^5^ cells was seeded in 24-well plates and cultivated with 1640 medium containing 10% newborn calf serum for 24 h. An amount of 1.25 µL of 20 µM miR-30a mimic/inhibitor or 1.25 µL of 20 µM TET1 siRNA was diluted with 50 µL 1× riboFECT^TM^ CP (RiboBio Co., Ltd., Guangzhou, China)buffer and incubated for 5 min at room temperature. A total of 5 µL riboFECT^TM^ CP reagents was added and incubated for 15 min at room temperature. The mixed liquid was added to 443.75 µL 1640 medium without 10% newborn calf serum. Cells were incubated with the mixed liquid for 48 h.

### 4.4. Quantitative Real-Time Polymerase Chain Reaction (qRT-PCR)

Total RNA was isolated using TRIzol reagent (Promega Corporation, Beijing, China). RNA quantity and quality were measured using the NanoDrop 2000 spectrophotometer (Thermo Fisher Scientific, Inc., Waltham, MA, USA), and RNA integrity was assessed using standard denaturing agarose gel electrophoresis. Complementary DNA synthesis was performed with the M-MLV reverse transcriptase kit (Promega Corporation) following the manufacturer’s instructions. qRT-PCR was performed with a SYBR green-based PCR master mix kit on a Rotor-Gene 3000 real-time PCR system (Corbett Robotics, Sydney, Australia). U6 served as an internal control.

### 4.5. Detection of Drp-1 Promoter Hydroxymethylation

The three methylation sites were predicted in the Drp-1 promoter region using Basic Local Alignment Search Tool analysis. Three primers were specifically designed for the three methylation sites as follows: F:5′-GGCTGGCTGTTCCCATCACTG-3′, R:5′-AAATGCTGCTTCGGCGTTCTC-3′; F:5′-TGGAGGCGCTAATTCCTGTCA-3′, R:5′-CTCTCACCTGCGTTCCCACTAC-3′; F:5′-AGAGGAGGAAGGAGGCGAACT-3′, R:5′-GCTTGTTTATGACAGGAATTAGCG-3′. DNA was ultrasonically treated for 4 min after extraction from the experimental cells. Subsequently, 400 µL dilution buffer and 60 µL protein G magnetic bead were added in turn. After 1 h, the mix system was placed on a magnetic frame for 10 min. An amount of 8 µg of 1 µg/µL mouse anti-5-hmc (Diagenode Inc., Liege, Belgium) was added at 4 °C overnight. The samples were prepared for analysis using qRT-PCR following the addition of 6 µg of 2 µg/µL mouse anti-IgG magnetic beads (Jackson ImmunoResearch Laboratories Inc., Lancaster, PA, USA).

### 4.6. Animal Model and Ethics Statement

C57BL/6 mice (8 weeks old) were obtained from the Model Animal Research Center of Nanjing University (Nanjing, China). All animal experiments were performed based on the regulations established by the Ethics Committee on Animal Experiments of Binzhou Medical University (196738, 01/09/2014) [39]. The mice were housed under a 12 h light/dark cycle and were allowed free access to food and water. The mice were then randomly divided into the following four groups (10 mice each): sham, BLM-treated (BLM), BLM+ negative control of miR-30a agomir (NC), and BLM+ miR-30a agomir groups. The miR-30a agomir dosage was 10 nmol for each mouse and was sprayed intothe lung twice per week for 4 weeks using a Penn-Century MicroSprayer (Penn-Century Inc.). On day 28, all mice were killed, and lung tissue sections were collected and immediately frozen in liquid nitrogen for further studies. The BLM animal model was administered with 5 mg/kg BLM dissolved in saline via a single intratracheal instillation under anesthesia as previously described [39,40].

### 4.7. Dual-Luciferase Assays

The WT and MT 3′UTR of TET1 containing miR-30a-binding site were synthesized by Obio Technology Co., Ltd. (Shanghai, China). The 293T cells were plated at 50%–60% confluence and transfected with miR-30 mimics and a 3′UTR luciferase vector using Lipofectamine™ 3000 (Thermo Fisher Scientific) transfection reagent based on the manufacturer’s instructions. At 48 h after transfection, luciferase assay was performed using a dual-luciferase reporter assay kit by following the manufacturer’s instructions.

### 4.8. Hematoxylin and Eosin (H&E)and Masson’s Trichrome Staining

Pulmonary tissues were fixed through inflation with 4% paraformaldehyde overnight, dehydrated in 70% ethanol, and embedded in paraffin wax. Sections with 4 mm thickness were prepared and stained with H&E or Masson’s trichrome staining (Sangon Biotech Inc., Shanghai, China) based on the manufacturer’s standard protocol. The degrees of microscopic interstitial fibrosis and collagen were graded as previously described [39].

### 4.9. Western Blot Analysis

Protein concentration was quantified using a bicinchoninic acid protein assay kit and boiled with the sample buffer in a water bath for 5 min. Protein samples were separated with 15% SDS-PAGE for 2 h and transferred onto a polyvinylidene difluoride membrane, which was subsequently blocked in 5% nonfat milk for 2 h. Blots were probed using the primary antibodies. The anti-Drp1 antibody was obtained from Santa Cruz Biotechnology. After washing with tris buffered saline tween, the horseradish peroxidase-conjugated secondary antibodies were added. Antigen–antibody complexes were visualized using enhanced chemiluminescence.

### 4.10. Statistical Analysis

Data were expressed as mean ± standard deviation (SD) from the indicated number of independent experiments. Statistical analysis was performed with SPSS 17.0 software (SPSS China Inc., Beijing, China) using one-way analysis of variance and Student’s *t*-test. *p* < 0.05 indicates statistically significant difference.

## Figures and Tables

**Table 1:** Characteristics and physiologies of idiopathic pulmonary fibrosis (IPF) patients and the normal individuals.

Characteristic	Normal	IPF	*p* Value
Number	46	46	/
Age (years)	68.29	68.17	/
Gender (Male/female)	23/23	23/23	/
FVC (% of predicted)	87.28	47.81	<0.01
TLC (% of predicted)	88.15	52.17	<0.01
DLCO (% of predicted)	89.17	63.75	<0.01
PaO_2_ (mmHg)	93.71	76.21	<0.01
PaCO_2_ (mmHg)	38.56	37.11	/
Smoking History (*n*)	4	15	<0.05

No statistical significance was observed among these characteristics because number, age, and gender of normal persons matched with those of IPF patients. FVC = forced vital capacity; TLC = total lung capacity; DLCO = diffusing capacity for carbon monoxide; Smoking history denotes subjects with >5 years of cigarette smoking. / Indicates no statistics.
